# PDGF-BB Deficiency in the Blood Serum from Aplastic Anemia Patients Affects Bone Marrow-Derived Multipotent Mesenchymal Stromal Cells

**DOI:** 10.3390/cells13221908

**Published:** 2024-11-18

**Authors:** Alena I. Dorofeeva, Irina N. Shipounova, Ksenia A. Nikiforova, Irina V. Galtseva, Larisa A. Kuzmina, Anton V. Luchkin, Zalina T. Fidarova, Elena A. Mikhailova, Elena N. Parovichnikova

**Affiliations:** National Medical Research Center for Hematology, Moscow 127167, Russia; nikiforova.k@blood.ru (K.A.N.); galtseva.i@blood.ru (I.V.G.); kuzmina.l@blood.ru (L.A.K.); luchkin.a@blood.ru (A.V.L.); fidarova.z@blood.ru (Z.T.F.); mikhailova.e@blood.ru (E.A.M.); parovichnikova.e@blood.ru (E.N.P.)

**Keywords:** hematopoietic microenvironment, multipotent mesenchymal stromal cells, aplastic anemia, blood serum, platelet-derived growth factor BB

## Abstract

Aplastic anemia (AA) is characterized by bone marrow (BM) aplasia and pancytopenia. BM stromal microenvironment is closely intertwined with hematopoietic cells by reciprocal regulation. It is still unclear how hematopoietic deficiency affects the bone marrow stroma of the AA patients. Multipotent mesenchymal stromal cells (MMSCs) are the progenitors of stromal cells. In vitro, proliferation rate of MMSCs of AA patients is decreased compared to those of healthy donors. This may be explained by the influence of pathological environmental condition in the patients’ BM. The aim of the study was to compare the effect of AA patients’ sera on healthy donor MMSCs to healthy donors’ sera and to elucidate the nature of their difference. Proliferation test showed 3-fold decrease in number of MMSCs after incubation in medium supplemented with AA patients’ sera compared to donors’ serum samples. The degree of this effect correlated with the severity of thrombocytopenia in patients. The decrease in cell number was not associated with cell death, as the number of apoptotic cells defined by flow cytometry did not differ between the groups. ELISA revealed a decreased level of PDGF-BB in the patients’ sera compared to donors’ serum samples (69 ± 5 pg/mL vs. 112 ± 21 pg/mL, respectively). The addition of recombinant PDGF-BB or healthy donor’s platelet lysate to the culture medium supplemented with AA patients’ serum restored its ability to support MMSCs growth. Thus, PDGF-BB deficiency is one of the environmental factors causing MMSCs damage in AA patients.

## 1. Introduction

Regulation of hematopoiesis by bone marrow (BM) stromal cells has been thoroughly described [1,2]. The earliest stromal precursors–multipotent stem cells (MSCs)–are localized at the outer wall of the capillaries and participate in formation of the vascular niche for the hematopoietic stem cells (HSCs). MSCs produce signaling molecules (CXCL12, KITLG, ANGPT1, VCAM1, SPP1) to maintain HSCs and regulate their functions [3]. MSCs can differentiate into osteoblasts, adipocytes and chondroblasts [4]. Osteoblasts form an osteoblastic niche where lymphoid precursors are maintained, and adipocytes in some cases can suppress hematopoiesis [5,6,7,8]. The ability of MSCs to differentiate and to produce signaling molecules is essential for maintenance of hematopoiesis and is impaired in some hematological diseases (e.g., acute myeloid leukemia, acute lymphoblastic leukemia, chronic myeloid leukemia, myelodysplastic syndrome, multiple myeloma) [9,10]. Another important property of MSCs is the ability to proliferate, as this enables MSCs to generate all types of stromal precursors. As a result, hematopoietic territory is formed. Two types of stromal precursors are isolated in vitro: multipotent mesenchymal stromal cells (MMSCs) and colony-forming units of fibroblasts [11,12]. Interaction with HSCs maintains the proliferative and differentiation potential of MSCs. However the influence of hematopoietic cells on the properties of stromal progenitors has not been sufficiently studied to date [13,14].

Aplastic anemia is a rare hematological disease characterized by BM aplasia and pancytopenia [15]. It seems to be an adequate model to study the influence of suppressed hematopoiesis on the properties of BM stroma. According to the severity of cytopenia, non-severe (NSAA), severe (SAA) and very severe AA (VSAA) are distinguished [16]. Although the pathogenesis of the disease is not fully understood, it is known that BM aplasia appears as a result of an autoimmune attack of T lymphocytes on HSCs [17]. Immunosuppressive therapy (IST), including antithymocyte globulin and cyclosporine A, is the main non-transplant approach for AA treatment [18]. Since a satisfactory response to IST is not achieved in all patients, and the complete response rate does not exceed 50%, other factors are expected to be involved in the pathogenesis of AA, such as, possibly, the impairment of the hematopoietic microenvironment. It was shown that AA patients’ BM-derived MMSCs retain both proliferative and differentiation potential and the ability to maintain hematopoietic precursors in vitro, which means that they are able to form functional hematopoietic niches [19,20]. However, the proliferation rate of patients’ MMSCs is reduced compared to those of donors [21,22]. For various reasons, AA patients differ in duration of cytopenia prior to the first visit to a hematologist. This means that BM microenvironment could be affected by pathological conditions for a considerable time. It had been shown that the delayed start of IST is associated with decreased response rate to IST in AA [23]. Long-term influence of pathological environmental conditions may affect stromal precursors in some patients, which may be the reason for poor response in these cases. We suggested the blood to reflect systemic changes in the body of AA patients.

This study aimed to investigate the influence of blood serum from AA patients on the properties of normal stromal cells. We incubated healthy donors’ MMSCs in the presence of AA patients’ or donors’ blood serum and demonstrated that AA patients’ serum was not able to support the proliferation of MMSCs compared to the serum obtained from healthy donors. We demonstrated that thrombocytopenia plays the key role in PDGF-BB deficiency in the AA patients’ serum.

## 2. Materials and Methods

### 2.1. Patients and Donors

The study included 43 AA patients (24 men, 19 women, 18–63 years old, median age 29 years). Among those 22 had NSAA, 16 had SAA and 5 had VSAA. Differential diagnosis of AA and assessment of disease severity were performed in accordance with international criteria [16]. Clinical characteristics of AA patients are listed in Supplementary Materials (Table S1). Blood serum and BM samples of all patients were obtained at the onset of the disease; in addition, serum samples of 12 patients were also obtained and analyzed 12 months after the beginning of IST. Protocol of IST included antithymocyte globulin and cyclosporine A; detailed protocol and criteria of response to therapy were previously described [24]. 

Blood serum samples from 8 healthy donors (4 men, 4 women, 18–43 years old, median age 35.5 years) were used as controls. Serum samples were frozen and stored at −70 °C until required.

Individual serum samples were used in some assays, while in other experiments we prepared a mixture of equal proportion of AA patients’ serum at the onset (n = 43; further indicated as AA MIX) and a mixture of donors’ serum (n = 6; further indicated as Donor MIX). For this, all serum samples were thawed at the same moment, and an equal amount of each sample was taken and pooled in the corresponding mixture (AA MIX or Donor MIX). Analysis of individual serum samples revealed uniform effect of serum from AA patients. Samples from donors also had minor deviations. Therefore, we decided to pool patients’ and donors’ samples correspondingly in order to decrease individual sample consumption.

Healthy donor MMSCs were obtained from BM samples of 19 healthy donors (11 men, 8 women, 13–61 years old, median age 23 years). The samples were collected during BM exfusion for allogeneic HSC transplantation in the Department of Hemoblastosis Chemotherapy and Bone Marrow and Hematopoietic Stem Cell Transplantation.

All blood serum and BM samples were obtained with informed consent signed in accordance with the Declaration of Helsinki. The study was approved by the Ethics Committee of the National Medical Research Center for Hematology, Ministry of Health, Russian Federation (ethical approval document reference number 173).

### 2.2. MMSCs Isolation and Culture

MMSCs were isolated out of 3–5 mL of BM samples in accordance with the standard method [25]. Isolated MMSCs met the requirements for this cell type [4] and were characterized by the immunophenotype CD73+CD90+CD105+CD34-CD45-CD14-HLA-DR-, as well as the ability to differentiate in the osteogenic and adipogenic direction, as previously demonstrated [25].

MMSCs were cultured in alpha-MEM (ICN, Laval, QC, Canada) supplemented with 10% fetal bovine serum (HyClone, Logan, UT, USA), 2 mM L-glutamine (ICN, Laval, QC, Canada), 100 U/mL penicillin (Synthesis, Moscow, Russia) and 50 µg/mL streptomycin (BioPharmGarant, Vladimir, Russia) at 37 °C and 5% CO_2_. The growth medium was replaced every 3–4 days. After reaching confluency, the cells were detached with 0.25% trypsin-EDTA solution (Paneco, Moscow, Russia), counted and seeded in T25 culture flask (Corning, New York, NY, USA) at the density of 4.0 × 10^3^ cells per cm^2^ of surface area.

### 2.3. Proliferation Assay

The influence of AA patients’ or donors’ blood serum on the growth of MMSCs was assessed using proliferation assay. MMSCs from one healthy donor were used for each experiment. Briefly, MMSCs at passage 1–5 were seeded at 800 cells per well in a 96-well plate (Sarstedt, Nümbrecht, Germany) in RPMI 1640 medium without phenol red (HyClone, USA) supplemented with 2 mM L-glutamine, 100 U/mL penicillin, 50 µg/mL streptomycin and 10% serum of each individual patient or donor (in 3 to 6 technical repeats). For some experiments, AA MIX and Donor MIX were used. Cells were incubated at 37 °C and 5% CO_2_ for a week. Then, the number of viable cells was accessed by CellTiter 96 AQueous Non-Radioactive Cell Proliferation Assay (Promega, Madison, WI, USA) according to manufacturer’s instruction. The resulting optical density (OD) values at λ = 492 nm were measured by Multiskan FC (Thermo Fisher Scientific, Waltham, MA, USA). At least three independent experiments with MMSCs from different donors were performed for each serum sample. To compare the results of different experiments, each OD value was normalized to the average OD value for all donors’ serum in a certain experiment, determining the MMSCs relative growth index. The growth index reflects the ratio of the number of MMSCs after incubation in a medium with the serum of a particular patient or donor to the average number of MMSCs from the same donor after incubation in a medium with the donors’ sera.

### 2.4. Apoptic Cell Detection

For apoptotic cell detection 70,000 MMSCs of the same healthy donor at passages 2–4 were seeded per well in a 6-well plate (Costar, Glendale, AZ, USA) in RPMI 1640 supplemented with 2 mM L-glutamine, 100 U/mL penicillin, 50 µg/mL streptomycin and 10% AA MIX or Donor MIX. After 1, 4, and 6 days of incubation at 37 °C and 5% CO_2_, cells were detached by trypsinization, and the proportion of apoptotic cells was determined by flow cytometry on BD FACS CANTO II using a BD Pharmingen FITC Annexin V Apoptosis Detection Kit I (BD Bioscience, Franklin Lakes, NJ, USA) according to manufacturer’s instruction. Data were processed using BDFACSDiva 6.1.3 software (BD Bioscience, USA) and Kaluza analysis software version 2.1 (Beckman Coulter, Brea, CA, USA). The resulting population of Annexin V+ propidium iodide–MMSC corresponds to early apoptotic cells; annexin V+ propidium iodide+ MMSC corresponds to late apoptotic cells. Five independent experiments on MMSCs of different donors were performed.

### 2.5. Population Doubling Time Determination

MMSCs prepared for apoptotic cell detection were counted and population doubling time (PDT) between 1st and 6th day of incubation in medium supplemented with AA MIX or Donor MIX was calculated by Equation (1):

PDT = t × ln(2)/(ln(N_6_) − ln(N_1_)) (1)

where t—MMSCs growing time in days,

N_6_—MMSCs number on 6th incubation day,

N_1_—MMSCs number on 1st incubation day. 

### 2.6. Analysis of the Effect of Recombinant Growth Factors and Human Platelet Lysate on MMSCs Proliferation

The following factors were used in the study: VEGFA165 (SCI-store, Moscow, Russia), PDGF-BB (Accellerate, Moscow, Russia), FGF2 (kindly provided by M. E. Gasparian, Senior Research Associate of Shemyakin–Ovchinnikov Institute of bioorganic chemistry RAS) [26]. Human platelet lysate (HPL) was prepared as described previously [27].

Briefly, MMSCs of the same healthy donor at passage 3–5 were seeded at 800 cells per well in a 96-well plate in RPMI 1640 supplemented with 2 mM L-glutamine, 100 U/mL penicillin, 50 µg/mL streptomycin and 10% AA MIX or Donor MIX. Each of the recombinant growth factors was added to the wells containing medium with AA MIX to final concentrations 0.1; 1; 10 or 100 ng/mL; HPL was added to final concentrations 0.001; 0.01; 0.1 or 1%. After incubation at 37 °C and 5% CO_2_ for a week, the number of viable cells was determined using the proliferation assay (see Section 2.3). Two to four independent experiments on MMSCs of different donors were performed for each factor.

### 2.7. Gene Expression Analysis

For gene expression analysis, RNA was extracted from MMSCs at passage 1. MMSCs of each patient and donor, whose serum was tested previously, were involved in this analysis. Cells were washed twice with Dulbecco’s Phosphate Buffered Saline (MP Biomedicals, Solon, OH, USA) and RNA were isolated by modified Chomczynski method [28] using TRIZOL (Ambion, Waltham, MA, USA). In subsequent reverse transcription reaction M-MLV reverse transcriptase (Promega, USA) and mixture of poly-T oligonucleotides and random hexamers in equal proportions as primers were used. Real-time polymerase chain reaction in TaqMan modification was carried out on 7500 amplificator (Applied Biosystems, Waltham, MA, USA). Target genes, as well as primers and probes sequences used are listed in the Supplementary Materials (Table S2). Relative gene expression level was determined by ∆∆Ct method as described previously [29]. For normalization, *GAPDH* and *BACT* were used as reference genes. 

### 2.8. Statistical Analysis

Statistical analysis was performed in GraphPad Prism 8 and RStudio 2023.12.1. The data distribution was determined using the Shapiro-Wilk test. The significance of differences between groups was assessed using the Welch corrected Student *t*-test or the Mann-Whitney U-test in the case of unpaired comparisons, and the paired Student *t*-test or the Wilcoxon test in case of paired comparisons. ANOVA followed with post hoc test was used to prove the differences. In correlation analysis according to the character of data distribution, the Pearson or Spearman R coefficient was calculated. Linear regression analysis was performed to prove the significance of the found parameters. The differences were considered statistically significant at *p* < 0.05. Results are presented as mean ± standard error of the mean; median values are indicated on scatter plots.

## 3. Results

### 3.1. The Ability of the AA Patients’ Blood Serum to Maintain the Growth of Healthy Donors’ MMSCs Is Impaired

**Figure 1:**
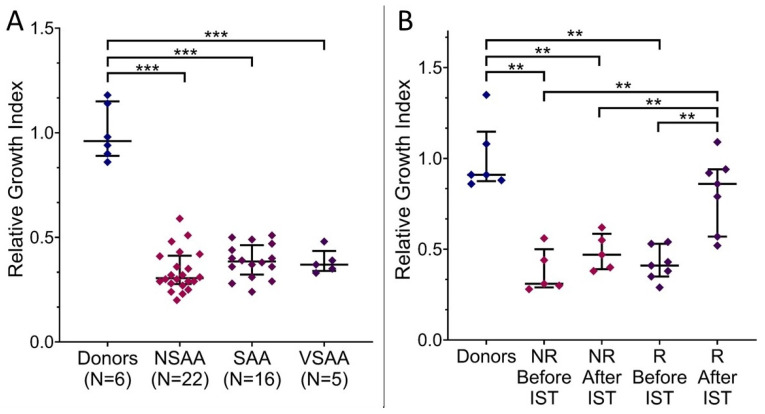
Relative growth index of healthy donors’ bone marrow (BM) derived multipotent mesenchymal stromal cells (MMSCs) after incubation in the medium containing 10% of aplastic anemia (AA) patients’ blood serum or donors’ serum: at the onset depending on the disease severity (**A**); at the onset and 12 months after the start of immunosuppressive therapy (IST) depending on the response to treatment (**B**). Each point represents the average value of relative growth index obtained in three independent experiments on MMSCs of three different donors. The horizontal line indicates the median, and the whiskers indicate the interquartile range. NSAA—non-severe AA, SAA—severe AA, VSAA—very severe AA, NR—non-responders, R–patients in remission. **, *** indicate significant differences (*p* < 0.01 and *p* < 0.001, respectively).

The growth index of MMSCs in the presence of serum from AA patients at the onset was reduced compared to that in the presence of serum from donors (0.36 ± 0.01 and 1.0 ± 0.05, respectively). There were no significant differences between groups of patients according to AA severity (Figure 1A), subsequent response to IST (Figure 1B) or disease duration (R = −0.02, see also Supplementary Materials, Table S3). Analysis of AA patients’ serum 12 months after the start of IST revealed significant differences between groups varied in response to IST (Figure 1B). MMSCs growth index increased significantly in the presence of responders’ blood serum (0.81 ± 0.08), in contrast to non-responders’ serum (0.48 ± 0.04).

Therefore, the ability of AA patients’ blood serum to maintain the growth of stromal precursors was impaired at the onset. This effect did not depend on the severity of the disease and the subsequent response to IST. A successful response to IST was associated with the restoration of the ability of AA patients’ serum to maintain the growth of stromal precursors.

### 3.2. Blood Sera from AA Patients Supress the Proliferation of Healthy Donors’ BM Derived MMSCs

**Figure 2:**
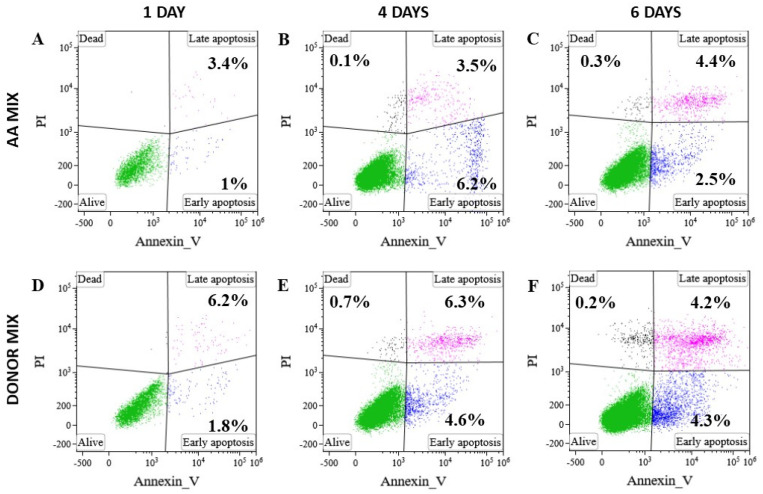
A representative case of flow cytometric determination of apoptotic MMSCs percentage in culture on days 1, 4, 6 of incubation in the presence of AA MIX (**A**,**B**,**C**, respectively) and Donor MIX (**D**,**E**,**F**, respectively). Annexin V+ PI–MMSCs correspond to early apoptotic cells; annexin V+ PI+ MMSCs correspond to late apoptotic cells. PI-propidium iodide.

The reduced number of viable MMSCs after incubation in the presence of AA patients’ blood serum indicates that serum components either induce cell death or suppress cell proliferation [30]. To assess the ability of AA patients’ serum to induce cell death, healthy donors’ MMSCs were incubated in the medium supplemented with a 10% of AA MIX or a 10% Donor MIX, and the percentage of cells in early and late apoptosis were estimated by flow cytometry on days 1, 4 and 6 of incubation (Table 1, Figure 2). There were no significant differences between cells incubated with the AA and Donor MIX. Cell number on days 1 and 6 of incubation with serum mixtures was counted. Two out of five MMSCs cultures from different donors in the presence of AA MIX did not proliferate at all. MMSCs of the remaining 3 cultures divided on average 3.4 times slower in the presence of AA MIX compared to Donor MIX (PDT was 7.1 ± 2.1 and 2.1 ± 0.3 days, respectively).

Therefore, the components of the serum from AA patients did not induce MMSCs death. AA patients’ blood serum inhibited or stopped the proliferation of healthy donors’ MMSCs, as there were individual differences in the response of MMSCs from different donors.

### 3.3. The Growth of Stromal Progenitors in the Presence of Serum of AA Patients Correlates with the Platelets Count in Their Blood Samples

To determine the factor(s) associated with the effect on the proliferation of stromal precursors in the serum from AA patients, we carried out a correlation analysis of the MMSCs relative growth index with more than 50 clinical and biochemical parameters of patients’ blood samples. All analyses that were performed for each blood sample are listed in Supplementary Materials (Table S4). Significant positive correlation (*p* < 0.001) was identified with the plateletcrit and with the platelet count in the blood on the day of sample collection (Spearman’s correlation coefficient R was 0.58 and 0.61, respectively). Plateletcrit is the platelet fraction volume in the blood and it depends on the platelet count (Spearman’s correlation coefficient R = 0.83). Thrombocytopenia is one of the diagnostic criteria for AA. At the time of blood sample collection, plateletcrit and platelet counts were below normal range in all patients except one, whose plateletcrit was slightly above the lower limit of the normal range. 

A majority of AA patients are transfusion-dependent, and their blood samples contain both their own and donor platelets. The production of their own platelets could be assessed by the minimum platelet count in a blood sample observed before transfusion. The minimum platelet count in the blood of patients did not correlate with the growth index of MMSCs in the presence of their serum (Pearson correlation coefficient R = 0.11). Thus, the growth index of MMSCs in the presence of AA patients’ serum at the onset correlated with the total platelet count in their blood samples, regardless of whether these formed elements are their own or donors’.

Linear regression analysis proved the leading role of platelet count and plateletcrit (multiple R^2^ = 0.5538, Table 2) in the inhibitory effect of AA serum on MMSCs growth.

Thus, the low number of platelets in the blood of AA patients determined its inhibitory effect on MMSCs growth.

### 3.4. The Level of Growth Factors Secreted by Platelets Is Reduced in Blood Sera from AA Patients

Platelets are the source of a large number of active molecules, including platelet-derived growth factor (PDGF) and vascular endothelial growth factor (VEGF), which are essential for the proliferation of stromal and endothelial cells [31,32]. Thrombocytopenia can lead to a deficiency of these factors in the blood and, as a consequence, to impaired proliferation of stromal cells in the presence of such serum. 

**Figure 3:**
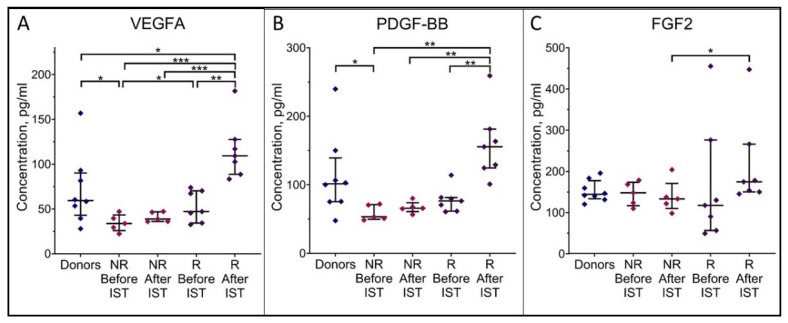
The growth factors level in the blood serum from AA patients and healthy donors: VEGFA (**A**), PDGF-BB (**B**), FGF2 (**C**). NR—non-responders, R—patients in remission, IST—immunosuppressive therapy. The horizontal line indicates the median, and the whiskers indicate the interquartile range. *, **, *** indicate significant differences (*p* < 0.05, *p* < 0.01 and *p* < 0.001, respectively).

ELISA revealed considerably decreased VEGFA and PDGF-BB levels in the blood sera of AA patients at the onset (45 ± 5 and 69 ± 5 pg/mL, respectively) compared to donors (71 ± 14 and 112 ± 21 pg/mL, respectively) (Figure 3). Moreover, in patients who did not subsequently respond to IST, VEGFA and PDGF-BB levels were significantly decreased (34 ± 4 and 59 ± 5 pg/mL, respectively) compared to donors (Figure 3A,B). A year after the start of IST, VEGFA and PDGF-BB levels had significantly increased in patients in remission (116 ± 12 and 159 ± 20 pg/mL, respectively) compared to non-responders (41 ± 2 and 67 ± 4 pg/mL, respectively) and to patients at the onset of AA. 

Thus, at the onset of AA, the patients’ blood serum VEGFA and PDGF-BB levels were decreased; however, they were restored in patients in remission 12 months after the start of IST. Perhaps, deficiency of these factors (or one of them) impaired the ability of AA patients’ blood serum to maintain the proliferation of healthy donors’ MMSCs.

Basic fibroblast growth factor (FGF2) is another stromal cell growth factor produced by various types of cells in the human organism [33]. FGF2 level in the blood serum of AA patients at the onset did not differ from the donors’ one (161 ± 30 and 153 ± 9 pg/mL, respectively, Figure 3C). A year after the start of IST, FGF2 level in patients in remission was significantly higher than in patients who did not respond to treatment (217 ± 42 and 139 ± 18 pg/mL, respectively). However, no significant differences between the patients at the onset and the donors were found.

Therefore, the decreased level of VEGFA and/or PDGF-BB in the AA patients’ blood serum at the onset may cause the suppression of healthy donors’ MMSCs proliferation.

### 3.5. The Ability of Blood Serum from AA Patients to Maintain the Proliferation of MMSCs Is Restored by the Addition of PDGF-BB, FGF2 or Human Platelet Lysate

**Figure 4:**
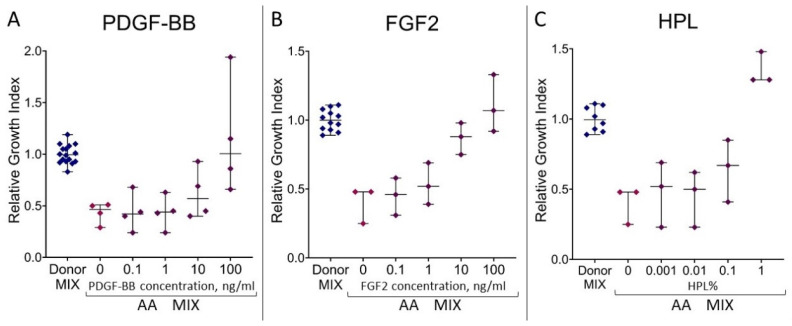
Relative growth index of healthy donors’ MMSCs after incubation in a medium containing 10% Donor MIX, 10% AA MIX without and with the addition of PDGF-BB (**A**), FGF2 (**B**) or healthy donor’s human platelet lysate (HPL) (**C**). The horizontal line indicates the median, and the whiskers indicate the interquartile range.

To find out whether the impaired ability of AA patients’ blood serum to maintain MMSCs’ proliferation is associated with a deficiency of growth factors, healthy donors’ MMSCs were incubated in a medium containing 10% AA MIX with the addition of recombinant growth factors or healthy donor’s HPL. Addition of 0.1–100 ng/mL VEGFA to the incubation medium had no effect on the growth index (0.35 ± 0.12 without VEGFA vs. 0.37 ± 0.13 with VEGFA). The addition of 100 ng/mL PDGF-BB, FGF2 or 1% HPL to the incubation medium led to an increase in the growth index from 0.42 ± 0.04 to 1.15 ± 0.28, 1.11 ± 0.12 and 1.35 ± 0.07, respectively. These results met the growth index values in the presence of 10% Donor MIX (1.00 ± 0.01), or even exceeded it (Figure 4). Although FGF2 addition turned out to rescue the MMSC growth, FGF2 level in AA patients’ blood serum was not decreased. Therefore, PDGF-BB shortage in blood serum from AA patients is a probable cause of the stromal insufficiency in those patients.

### 3.6. AA Patients’ MMSCs Revealed Altered Expression of Growth Factor Receptor Genes, Which Negatively Correlated with the Platelet Count in Their Blood

**Figure 5:**
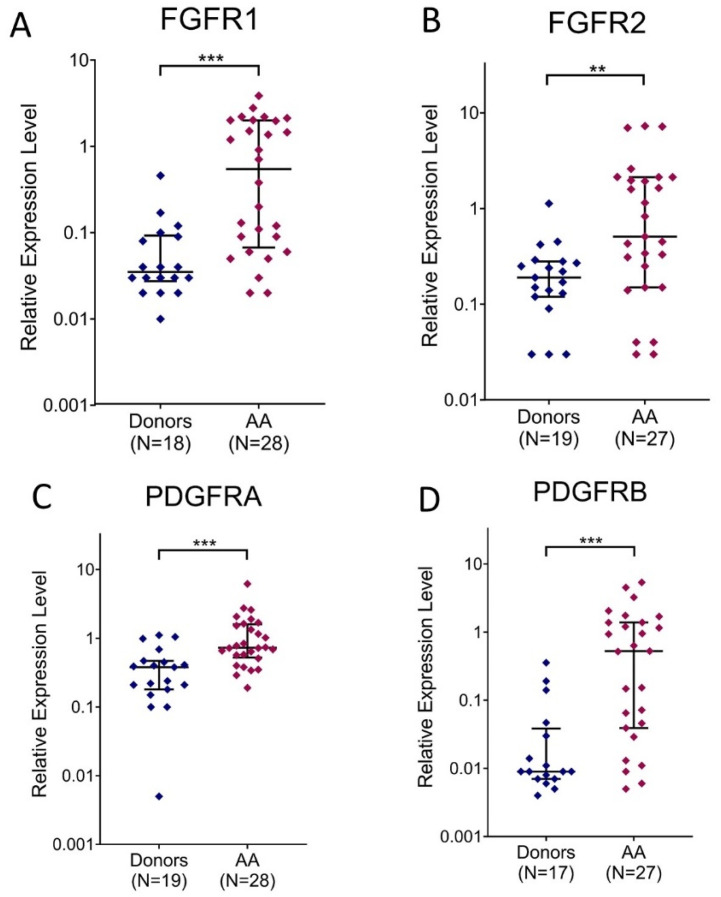
Relative gene expression level in MMSCs from the BM of healthy donors and AA patients of FGFR1 (**A**), FGFR2 (**B**), PDGFRA (**C**), PDGFRB (**D**). The horizontal line indicates the median, and the whiskers indicate the interquartile range. The data are presented in logarithmic scale. **, *** indicate significant differences (*p* < 0.01 and *p* < 0.001, respectively).

In the organism of AA patients, BM stromal cells exist in contact with insufficient blood sera for considerable time. To find out whether it affects their gene expression profile, the expression levels of growth factors (*VEGFA* and *FGF2*) and growth factor receptor genes (*FGFR1*, *FGFR2*, *PDGFRA* and *PDGFRB*) were analyzed in MMSCs of 28 AA patients, whose serums had been analyzed in this study. The expression levels of all analyzed growth factor receptor genes were significantly higher in MMSCs from AA patients than in those from donors (Figure 5).

Correlation analysis revealed low to moderate negative correlation between all analyzed growth factor receptor genes expression in MMSCs from patients and minimal platelet count in their blood. Significant correlation (*p* < 0.05) was found for *FGFR1* and *PDGFRA* gene expression (R = −0.47 and R = −0.40, respectively), non-significant correlation–for *FGFR2* and *PDGFRB* (for both R = −0.31, *p* = 0.11).Thus, decreased platelet production in AA patients is associated with increased expression of growth factor receptor genes in stromal precursor cells.

Moderate yet significant negative correlation was found between *VEGFA* gene expression level in MMSCs from patients and the platelet count in their blood (R = −0.56, *p* < 0.01). This may indicate compensatory activation of *VEGFA* transcription in BM stromal cells depending on the severity of thrombocytopenia and should be further investigated.

## 4. Discussion

We have shown that thrombocytopenia in AA is associated with a decreased PDGF-BB blood serum level. As a result, such serum loses proper ability to maintain the proliferation of healthy donors’ BM-derived MMSCs. PDGF-BB is a local regulator of cell functions. Normally in the BM, PDGF-BB is produced by immature hematopoietic precursors and megakaryocytes, which are significantly reduced in number in AA [34]. This should lead to a decreased PDGF-BB level not only in the blood, but also in the BM of AA patients. Therefore, AA patients’ BM stromal cells function under deficiency of the growth factor, which is essential for their proliferation. This affects proliferative properties of MMSCs and, consequently, their ability to maintain normal hematopoiesis. Perhaps this phenomenon has a role in the absence or insufficient response to IST in some cases. Indeed, BM MMSCs from AA patients at the onset differ from those from healthy donors in a reduced proliferation rate [19]. Moreover, as we have shown, MMSCs from AA patients are characterized by increased expression levels of growth factor receptor genes, which negatively correlates with platelet count in the blood of corresponding patients and indicates a disruption in growth factor signaling. This confirms the suggestion that in AA patients’ BM, MMSCs are affected by the revealed growth factor deficiency. The observed upregulation of the expression of growth factor receptor genes may be a compensatory reaction of the cells to the low concentration of the growth factors in the serum.

We have shown that the ability of the blood serum from AA patients to maintain the proliferation of MMSCs depends on the total platelet count in their blood samples, regardless of whether they are derived from the host or a donor. Indeed, any platelets, regardless of their origin, secrete growth factors, and platelet transfusions apparently compensate at least partially for the deficiency of growth factors in AA patients’ blood. This explains why there is no difference in the growth index of MMSCs after incubation in the presence of serum of AA patients with various forms of the disease.

Platelets and megakaryocytes are the sources of another growth factor, VEGFA [35]. The VEGFA level was decreased in blood serum from AA patients at the onset and was restored after achieving remission, which is consistent with other research [36]. It is known that a decreased VEGFA level in the blood from AA patients at the onset is associated with a decreased microvessel density in the BM, and its restoration in patients after successful therapy is associated with the recovery of VEGFA level [36]. VEGFA is necessary for the proliferation of endothelial cells and for angiogenesis. One study reported its role in the restoration of the proliferation rate of MMSCs from AA patients in vitro [37]. However, in our work the addition of VEGFA to a medium containing 10% of serum from AA patients did not restore the proliferation of MMSCs from healthy donors. A decreased level of growth factors VEGFA and PDGF-BB secreted by platelets in AA probably impairs the proliferation of both endothelial and stromal cells. Since these cell types participate in the BM vascular niche formation, this may lead to disruption of this microenvironment. The influence of BM aplasia and thrombocytopenia on the properties of endothelial cells of the vascular niche in patients with AA should be further investigated.

FGF2 is another stromal cell growth factor, secreted by various types of cells. Although FGF2 level in the blood serum from AA patients at the onset was not altered compared to donors, the addition of this factor to incubation medium containing 10% of serum from AA patients compensated for the PDGF-BB deficiency and restored MMSCs proliferation. This may be due to the activation of the same cell signaling cascades by both factors. Indeed, the binding of PDGF-BB or FGF2 to their corresponding receptors leads to the activation of common signaling pathways: RAS/MAP Kinase, PI3 Kinase/AKT, PLCγ and JAK/STAT pathways [31,33]. These signaling pathways control not only cell proliferation but also apoptosis, migration, chemotaxis and metabolism of stromal cells. PDGF-BB deficiency probably also leads to dysregulation of all these processes in stromal precursors in BM of AA patients. Studying the influence of a growth factors deficiency on properties of MMSCs will expand our understanding of niche functioning in normal and pathological conditions. 

The described MMSCs damage may contribute to the absence or insufficiency of response to IST in some cases. It remains to be determined whether the proliferation rate and the expression level of growth factor receptor genes in stromal progenitors are restored in remission and whether activation of the PDGF signaling can restore the proliferative properties of stromal cells in patients who did not respond to therapy.

To summarize, we have shown that thrombocytopenia in AA led to PDGF-BB deficiency in patients’ blood serum, which resulted in suppressed BM stromal cells proliferation. Decreased platelet production is characteristic of not only AA but also of different hematological diseases, including myelodysplastic syndrome, leukemias, thrombocytopenias, etc. Further studies of the influence of the duration of thrombocytopenia on the properties of stromal progenitors may improve our understanding of the niche functioning at various hematological diseases.

## Figures and Tables

**Table 1:** The proportion of apoptotic cells in the MMSCs cultures from healthy donors incubated in the presence of AA MIX or Donor MIX. Data are presented as mean ± standard error of mean (n = 5).

MMSCs in early apoptosis, %			
Days of incubation	1	4	6
AA MIX	2.2 ± 0.4	2.7 ± 1.0	1.6 ± 0.3
Donor MIX	3.3 ± 0.4	2.6 ± 0.6	2.0 ± 0.6
MMSCs in late apoptosis, %			
Days of incubation	1	4	6
AA MIX	3.5 ± 1.0	4.1 ± 0.4	5.7 ± 1.3
Donor MIX	7.4 ± 2.8	3.9 ± 0.7	4.8 ± 0.4

**Table 2:** Summary of the linear regression parameters.

Growth Index~Platelet Count + Plateletcrit					
Residuals:					
Min	1Q	Median	3Q	Max	
−0.099552	−0.036585	−0.00846	0.037951	0.145072	
Coefficients:					
	Estimate	Std. Error	t value	Pr(>|t|)	Signif. codes
(Intercept)	0.1395107	0.055883	2.496	0.0168	*
platelets count	0.0023182	0.000343	6.756	4.11 × 10^−8^	***
plateletcrit	0.0118075	0.004562	2.588	0.0134	*
Residual standard error: 0.06155 on 40 degrees of freedom					
Multiple R-squared: 0.5538, Adjusted R-squared: 0.5315					
F-statistic: 24.83 on 2 and 40 DF, *p*-value: 9.768 × 10^−8^					

*, *** indicate significant differences (*p* < 0.05 and *p* < 0.001, respectively).

## Data Availability

Dataset available on request from the authors.

## Supplementary Materials

The following supporting information can be downloaded at https://www.mdpi.com/article/10.3390/cells13221908/s1, Table S1: Characteristics of AA patients included in the study; Table S2: Nucleotide sequences of primers and probes used for gene expression analysis; Table S3: Relative growth index of healthy donors’ MMSCs in the presence of serum of AA patients differing in the duration of the disease; Table S4: Parameters of patients’ blood samples tests included in statistical analysis.
