# Supplementary material for: PDGF-BB Deficiency in the Blood Serum from Aplastic Anemia Patients Affects Bone Marrow-Derived Multipotent Mesenchymal Stromal Cells
Source: Cells. 2024 Nov 18;13(22):1908. doi: 10.3390/cells13221908 (PMC11592413; doi:10.3390/cells13221908)
Supplement: Supplementary file 1 [file cells-13-01908-s001.zip › cells-3306195_S1.pdf]

Table S1. Characteristics of AA patients included in the study

№№	Gender	Age, y.o.	Diagnosis	Hemoglobin, g/L	Platelets x 10^9/L	Neutrophils x 10^9/L
1	m	22	SAA	49	12	0.18
2	f	25	NSAA	89	19	0.86
3	f	27	NSAA	52	15	0.77
4	m	49	SAA	62	18	0.48
5	m	20	SAA	94	27	0.46
6	m	23	SAA	66	24	0.92
7	m	40	NSAA	74	18	0.68
8	m	51	NSAA	78	7	1.39
9	f	39	NSAA	79	24	1.4
10	f	32	NSAA	74	15	1.23
11	f	29	SAA	72	29	0.48
12	m	20	SAA	66	3	0.13
13	m	37	NSAA	49	7	0.77
14	m	21	NSAA	100	32	1.49
15	f	20	SAA	89	33	0.36
16	f	18	SAA	73	15	0.43
17	f	29	NSAA	74	15	0.96
18	f	31	NSAA	89	14	0.4
19	m	24	NSAA	70	22	1.08
20	m	35	NSAA	78	10	0.7
21	f	35	SAA	68	12	1.95
22	m	49	NSAA	79	8	2.04
23	m	26	VSAA	65	3	0.11
24	f	49	SAA	59	11	0.27
25	m	28	VSAA	71	17	0.04
26	f	46	NSAA	60	5	0.4
27	m	26	NSAA	81	9	0.65
28	f	43	SAA	60	5	0.09
29	f	31	NSAA	76	10	0.7
30	f	28	SAA	61	12	0.65
31	m	26	SAA	60	3	0.3
32	m	63	SAA	66	15	0.23
33	f	22	NSAA	74	6	1.99
34	m	36	NSAA	68	7	0.63
35	m	19	VSAA	41	13	0.17
36	f	31	NSAA	90	14	0.71
37	m	26	NSAA	74	29	0.74
38	m	28	VSAA	72	16	0.03
39	m	22	SAA	66	12	0.34
40	f	31	NSAA	86	7	1.18
41	f	57	SAA	70	9	0.14
42	m	21	VSAA	60	8	0.09
43	m	33	NSAA	84	5	1.76
