# Supplementary material for: PDGF-BB Deficiency in the Blood Serum from Aplastic Anemia Patients Affects Bone Marrow-Derived Multipotent Mesenchymal Stromal Cells
Source: Cells. 2024 Nov 18;13(22):1908. doi: 10.3390/cells13221908 (PMC11592413; doi:10.3390/cells13221908)
Supplement: Supplementary file 1 [file cells-13-01908-s001.zip › cells-3306195_S2.pdf]

Table S2. Nucleotide sequences of primers and probes used for gene expression analysis

Gene	Primer/Probe	Nucleotide Sequence
ACTB	forward	CAACCGCGAGAAGATGACC
	reverse	CAGAGGCGTACAGGGATAGC
	probe	ROX-AGACCTTCAACACCCCAGCCATGTACG-BHQ2
FGF2	forward	GAAGAGCGACCCTCACATCAAG
	reverse	TCCGTAACACATTTAGAAGCCAGTA
	probe	FAM-TCATAGCCAGGTAACGGTTAGCACACACTCCT-RTQ1
FGFR1	forward	CAGAATTGGAGGCTACAAGG
	reverse	TGATGCTGCCGTACTCATTC
	probe	FAM- CATCATAATGGACTCTGTGGTGC-RTQ1
FGFR2	forward	CTCATTATGGAAAGTGTGGTC
	reverse	TGGGCCGGTGAGGCGATC
	probe	FAM- CAGGTGGTACGTGTGATTGATGGA -RTQ1
GAPDH	forward	GGTGAAGGTCGGAGTCAACG
	reverse	TGGGTGGAATCATATTGGAACA
	probe	ROX-CTCTGGTAAAGTGGATATTGTTGCCATCA-BHQ2
PDGFRA	forward	TGGCTAAGAATCTCCTTGGA
	reverse	ACCAGGACAATAAGTGAGATG
	probe	FAM-CAATCACCAACAGCACCAGGACT-RTQ1
PDGFRB	forward	CTCCCTTATCATCCTCATCA
	reverse	TCCACGTAGATGTACTCATG
	probe	FAM-TCACAGACTCAATCACCTTCCATC-RTQ1
VEGFA	forward	AGGCGAGGCAGCTTGAGTTA
	reverse	ACCCTGAGGGAGGCTCCTT
	probe	FAM-CCTCGGCTTGTCACATCTGCAAGTACGT-RTQ1
