# Supplementary material for: PDGF-BB Deficiency in the Blood Serum from Aplastic Anemia Patients Affects Bone Marrow-Derived Multipotent Mesenchymal Stromal Cells
Source: Cells. 2024 Nov 18;13(22):1908. doi: 10.3390/cells13221908 (PMC11592413; doi:10.3390/cells13221908)
Supplement: Supplementary file 1 [file cells-13-01908-s001.zip › cells-3306195_S3.pdf]

Table S3. Relative growth index of healthy donors' MMSCs in the presence of serum of AA patients differing in the duration of the disease

Duration of the disease before treatment , months	1-6<br>(N=19)	7-8<br>(N=15)	9-43<br>(N=8)
Relative growth index	0.35 ± 0.02	0.37 ± 0.02	0.36 ± 0.05
