# Supplementary material for: PDGF-BB Deficiency in the Blood Serum from Aplastic Anemia Patients Affects Bone Marrow-Derived Multipotent Mesenchymal Stromal Cells
Source: Cells. 2024 Nov 18;13(22):1908. doi: 10.3390/cells13221908 (PMC11592413; doi:10.3390/cells13221908)
Supplement: Supplementary file 1 [file cells-13-01908-s001.zip › cells-3306195_S4.pdf]

Table S4. Parameters of patients' blood samples tests included in statistical analysis.

General blood test
% segmented neutrophils, hemoglobin, red blood cell count, hematocrit, mean corpuscular volume, plateletcrit, % segmented eosinophils, white blood cell count, mean corpuscular hemoglobin, mean corpuscular hemoglobin concentration, red cell distribution width, platelet count, large platelet fraction, platelet distribution width, mean platelet volume, % neutrophils, neutrophil count, % lymphocytes, lymphocyte count, % monocytes, monocyte count, % eosinophils, eosinophil count, % bands neutrophils, % segmented basophils, % reticulocyte, reticulocyte count, erythrocyte sedimentation rate, % basophils, basophil count.
Biochemical blood test
Total protein, albumin, urea, creatinine, calcium, potassium, sodium, total bilirubin, direct bilirubin, indirect bilirubin, gamma-glutamyltransferase, alkaline phosphatase, aspartate aminotransferase, alanine aminotransferase, lactate dehydrogenase, uric acid, chloride, phosphorus, magnesium, serum iron, C-reactive protein, globulin, albumin/globulin ratio, cholesterol, triglyceride.
Monitoring of anemia test
Latent iron-binding capacity, erythropoietin, ferritin, folates, antibodies to intrinsic factor, vitamin B12, transferrin, total iron-binding capacity, transferrin saturation with iron.
